# Circulating miRNAs in the Plasma of Post-COVID-19 Patients with Typical Recovery and Those with Long-COVID Symptoms: Regulation of Immune Response-Associated Pathways

**DOI:** 10.3390/ncrna10050048

**Published:** 2024-09-02

**Authors:** Anna M. Timofeeva, Artem O. Nikitin, Georgy A. Nevinsky

**Affiliations:** 1 SB RAS Institute of Chemical Biology and Fundamental Medicine, 630090 Novosibirsk, Russia; 2 Faculty of Natural Sciences, Novosibirsk State University, 630090 Novosibirsk, Russia

**Keywords:** miRNA, COVID-19, long COVID, antibodies, catalytic antibodies, SARS-CoV-2, miRNA, RNA hydrolysis, miR-200c-3p, miR-766-3p, miR-142-3p, pathways, regulatory network, rheumatologically

## Abstract

Following the acute phase of SARS-CoV-2 infection, certain individuals experience persistent symptoms referred to as long COVID. This study analyzed the patients categorized into three distinct groups: (1) individuals presenting rheumatological symptoms associated with long COVID, (2) patients who have successfully recovered from COVID-19, and (3) donors who have never contracted COVID-19. A notable decline in the expression of miR-200c-3p, miR-766-3p, and miR-142-3p was identified among patients exhibiting rheumatological symptoms of long COVID. The highest concentration of miR-142-3p was found in healthy donors. One potential way to reduce miRNA concentrations is through antibody-mediated hydrolysis. Not only can antibodies possessing RNA-hydrolyzing activity recognize the miRNA substrate specifically, but they also catalyze its hydrolysis. The analysis of the catalytic activity of plasma antibodies revealed that antibodies from patients with long COVID demonstrated lower hydrolysis activity against five fluorescently labeled oligonucleotide sequences corresponding to the Flu-miR-146b-5p, Flu-miR-766-3p, Flu-miR-4742-3p, and Flu-miR-142-3p miRNAs and increased activity against the Flu-miR-378a-3p miRNA compared to other patient groups. The changes in miRNA concentrations and antibody-mediated hydrolysis of miRNAs are assumed to have a complex regulatory mechanism that is linked to gene pathways associated with the immune system. We demonstrate that all six miRNAs under analysis are associated with a large number of signaling pathways associated with immune response-associated pathways.

## 1. Introduction

MiRNAs are a class of noncoding RNAs that regulate the expression of numerous genes through degradation, translational repression, or activation of mRNAs [1] as they are involved in various biological processes such as regulation of immune response, immune cell differentiation, metabolism, apoptosis, cell cycle, and oncogenesis [2]. Numerous studies have demonstrated that miRNAs can be released into extracellular fluids [3]. Hence, extracellular miRNAs represent promising biomarkers for early diagnosis and prognosis of various diseases, as well as targets for therapy [4]. Moreover, miRNA-based drugs are expected to become the next generation of drugs for the treatment and prevention of various human diseases [5]. 

Various diseases, including viral infections, cause significant changes in the expression profile of miRNAs [6,7]. Currently, a large number of differentially expressed miRNAs associated with COVID-19 have been identified [8,9]. These studies identify miRNAs associated with severe COVID-19, and gene regulatory pathways to understand the underlying biological processes associated with COVID-19 [10]. For example, Nicoletti et al. [11] identified 18 plasma miRNAs that are differentially expressed in COVID-19 patients and controls, including miR-4433b-5p, miR-320b, and miR-16-2-3p. Expression of the miR-320 family (320a-3p, 320b, 320c, and 320d) is increased in patients with COVID-19 compared to healthy controls [12], and lower miR-320 expression is associated with death in patients [11,13]. Changes in the expression level of some miRNAs, such as miR-142-3p, miR-146b-5p, miR-148a-3p, miR-200c-3p, miR-370, miR-378a-3p, miR-483, miR-1246, and others, have been described during the acute period of SARS-CoV-2 infection [14,15,16]. Several miRNAs, such as miR-21, miR-143, miR-122, miR-133, miR-155, miR-208a, miR-499, miR-625, and others, are associated with disease severity [8,16,17]. Data on the expression of some miRNAs in COVID-19 are contradictory (e.g., miR-4433b-5p, miR-16-2-3p) [12,14,18,19], which is probably due to differences in the patient sample. 

MiRNAs regulating the expression of viral genes are interesting. Several studies indicate that the interaction of host miRNAs with viral genes limits viral replication [20]. MiRNAs capable of targeting various regions of the SARS-CoV-2 genome, such as miR-197-5p and miR-18b-5p, have been identified [21,22]. 

MiRNA-mediated regulation has been shown not only for higher eukaryotes. To date, miRBase reports 30 viral miRNAs encoded by RNA viruses [23,24,25,26]. Several studies have demonstrated miRNAs originating from the SARS-CoV-2 genome [27,28,29]. 

Although most patients with COVID-19 fully recover, some experience lingering symptoms that may be related to previous SARS-CoV-2 infection (long COVID) [30]. The clinical manifestations associated with long-term COVID are incredibly heterogeneous and involve the respiratory system, gastrointestinal tract, joints, nervous system, endocrine system, etc. [31,32]. During the course of COVID-19, an important proportion of cases suffer from severe pneumonia and tend to have long-term sequelae [33]. Ongoing fibrosis during the recovery period results in decreased diffusion capacity of the lung [34]. There are a significant number of reports of patients with demyelinating pathologies such as Guillain–Barré syndrome, Miller–Fisher syndrome and others [35]. There is growing published data on a wide range of autoimmune diseases associated with SARS-CoV-2 [36,37]. To date, it has been confirmed that viral infections are associated with the development of various rheumatological diseases [38,39]. Medical experts have provided detailed descriptions and characterizations of certain cases, but the specific molecular mechanisms remain insufficiently researched. 

Dysregulation of some miRNAs was suggested to lead to long COVID [40]. For example, miR-34a, contained in circulating extracellular vesicles, is associated with the risk of diabetes after COVID-19 infection [41]. Some miRNAs are involved in the pathogenesis of thromboembolic complications in COVID-19 [42]. For example, decreased expression of miR-103a, miR-145, and miR-885 and increased expression of miR-424 and miR-320 were found in a group of patients with a high frequency of thrombotic and ischemic events [43,44]. It has been shown that the concentration of exosomal miR-145 and miR-885 significantly correlates with D-dimer levels [45]. In some patients, SARS-CoV-2 causes an excessive immune response, resulting in a cytokine storm [46]. Some miRNAs are involved in the regulation of mediators associated with inflammation and appear to be important in some inflammatory diseases [47]. Significantly decreased miRNA-106a and miRNA-20a expression are associated with increased IL-10, TNF-α, INF-γ, and TLR-4 levels in COVID-19 patients [12,14,48]. This is not surprising since these miRNAs target proinflammatory cytokine genes (e.g., TNF, CCL2, CXCL9, IL10) and cytokine and chemokine receptors (IL1R1, IL2RA, IFNAR2) [49]. 

In light of the COVID-19 pandemic, extensive research has been dedicated to studying the immune response to the SARS-CoV-2 virus. Involved in the immune response to viral infection are catalytically active antibodies. These antibodies exhibit a dual function of antigen binding and catalyzing the hydrolysis of particular substrates, including proteins, nucleic acids, and polysaccharides [50,51]. To illustrate, antibodies displaying proteolytic activity were reported in several studies [52,53]. Additionally, there have been advancements in the creation of monoclonal antibodies that can directly hydrolyze the RNA of coronavirus and influenza virus [54,55,56,57]. Previous studies have described the capability of catalytically active antibodies to hydrolyze miRNAs in the context of autoimmune and neurological diseases [58,59,60,61]. 

Our study focused on evaluating the expression patterns of ten miRNAs in the plasma of COVID-19 patients, differentiating between those who had recovered without any complaints and those who continued to experience lingering rheumatological symptoms associated with long COVID. The focus of this study was to analyze the role of IgG in miRNA hydrolysis among COVID-19 patients. We proposed the idea that the presence of catalytically active antibodies with a specific affinity for particular miRNAs might lead to changes in the plasma concentration of these miRNAs through their hydrolysis. Also, among a large number of miRNA signaling pathways, pathways associated with the immune response have been identified.

## 2. Results

### 2.1. General Characteristics of the Patient Groups

Following the acute phase of SARS-CoV-2 infection, certain individuals were found to experience persistent symptoms referred to as long COVID [62]. Numerous respiratory, cardiovascular, autoimmune, neurological, and psychiatric diseases, along with their associated symptoms, have been documented in relation to long COVID [63,64,65,66,67,68]. Additionally, there are reports on the incidence of autoimmune diseases after SARS-CoV-2 infection [69,70,71]. The focus of this study was to identify and analyze a particular group of patients who presented complaints related to rheumatological conditions, known as the Lon group. We established a cohort of COVID-19 survivors who exhibited no post-recovery symptoms (Cov) and a control group of healthy individuals who had not contracted COVID-19 (Neg). The collection of blood plasma from COVID-19 patients occurred during the circulation of the Wuhan strain of SARS-CoV-2. Between summer 2020 and spring 2021, the epidemic in Russia was characterized by the spread of three lineages: B.1.1.7, B.1.1.317, and a sublineage of B.1.1 including B.1.1.397 [72]. The study did not include subjects vaccinated against SARS-CoV-2.

### 2.2. Analysis of miRNA Concentration in the Plasma of Patients

The concentrations of ten miRNAs (miR-l7f-5p, miR-146b-5p, miR-148a-3p, miR-200c-3p, miR-378a-3p, miR-9-5p, miR-766-3p, miR-3125, miR-4742-3p, and miR-142-3p) were studied in the blood plasma of patients in three groups. For this purpose, a reverse transcription method using appropriate stem-loop (SL) primers followed by real-time PCR was used. A calibration line was constructed using a standard with a known concentration to quantify the miRNA concentration. A standard was prepared using synthetic miRNA miR-148a-3p at concentrations of 10^−1^ ng/μL, 10^−3^ ng/μL, and 10^−5^ ng/μL. The quantification of cycle (Cq) values were calculated based on the amplification curves for different concentrations of synthetic miRNA. These values were used to create a calibration curve, and an equation was derived to accurately calculate the concentration of the researched miRNAs.

**Figure 1:**
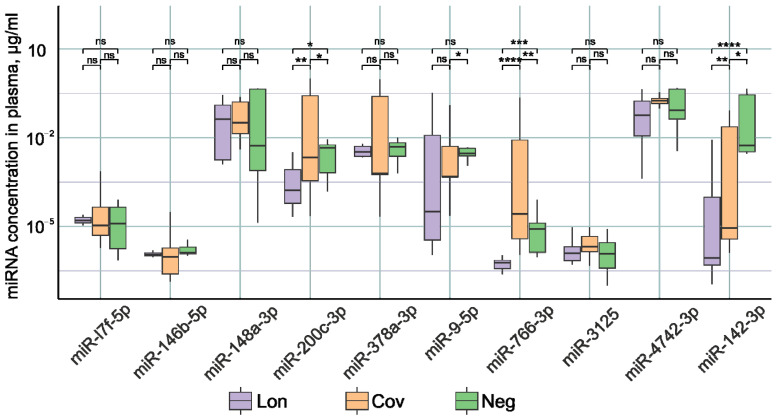
Examination of miRNA levels in the plasma of individuals within the three study cohorts: **Lon** refers to patients who experienced persistent rheumatologic symptoms alongside COVID-19, **Cov** denotes patients who successfully recovered from COVID-19 without any lingering complaints, and Neg represents disease-free donors. *—*p*-value < 0.05; **—*p*-value < 0.01; ***—*p*-value < 0.001, ****—*p*-value < 0.0001, ns—not significant.

Figure 1 demonstrates statistically significant differences in the concentrations of miR-200c-3p, miR-766-3p, and miR-142-3p between patient groups. In the Lon group, there was a significant reduction in the expression of miR-200c-3p, miR-766-3p, and miR-142-3p compared to the other two groups. It was previously shown that in the acute phase of COVID-19 infection, patients experience the decreased expression of miR-142-3p [14,15,73]. Decreased miR-142-3p expression is associated with the inflammatory process [74,75,76]. Our results demonstrate that miR-142-3p levels remain lower after recovery compared to healthy patients. It is likely that normalization of miR-142-3p expression requires a longer time than 3–6 months after recovery.

The highest concentration of miR-200c-3p and miR-766-3p was identified in the group of patients with no complaints after COVID-19. In the post-recovery period, we observed a reduced expression of these miRNAs. However, an increase in miR-200c-3p was reported in studies undertaken during the acute period of SARS-CoV-2 infection [77]. Moreover, the increase in the severity of the disease correlates with an increase in the expression of miR-200c-3p [78,79]. miR-200c-3p directly targets the 3′-untranslated region of ACE2 mRNA, resulting in decreased ACE2 expression [80]. miR-200c-3p expression is induced via the NF-κB pathway during infection. In particular, the NF-κB signaling pathway is hyperactivated during SARS-CoV-2 infection, leading to the overexpression of miR-200c-3p in COVID-19 patients with active disease. This leads to a decrease in ACE2 protein levels [81]. Decreased ACE2 expression leads to decreased susceptibility to SARS-CoV-2 entry into the host cell [82]. Thus, miR-200c-3p during COVID-19 infection by regulating ACE2 expression reduces further virus penetration into the cell, limits the spread of the virus, and has a positive effect on the recovery process. It is logical to assume that after recovery, normalization of ACE2 expression is necessary; therefore, miR-200c-3p expression decreases, which leads to an increase in ACE2 protein production until normal levels are reached. This is confirmed by our results of decreased miR-200c-3p expression in recovered patients from 3–6 months after COVID-19.

Data in the literature on the role of miR-766-3p in COVID-19 are scarce. Downregulation of this miRNA has been described in acute respiratory distress syndrome patients [19]. Our results demonstrate that miR-766-3p levels remain reduced 3–6 months after recovery.

### 2.3. Analysis of the Catalytic Activity of Antibodies in the Hydrolysis of miRNAs

The affinity chromatography method utilizing immobilized G-protein allows for the extraction of antibody preparations from different biological fluids, guaranteeing the absence of any protein or enzyme impurities [83]. In this study, IgG preparations were isolated from the blood plasma of the patients of the three study groups. To substantiate the specific catalytic function attributed to immunoglobulins, we employed the methodologies proposed by C. Paul [84] and further developed by the group of G. A. Nevinsky [50], in a similar way as described in [53]. 

**Figure 2:**
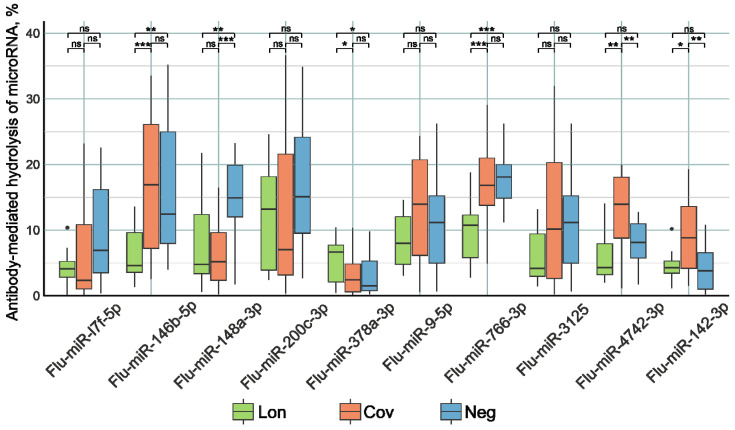
The comparative activity of Flu-miRNA hydrolysis by plasma IgG preparations of patients in the three study groups. Lon refers to patients who experienced persistent rheumatologic symptoms alongside COVID-19, Cov denotes patients who successfully recovered from COVID-19 without any lingering complaints, and Neg represents disease-free donors. *—*p*-value < 0.05; **—*p*-value < 0.01; ***—*p*-value < 0.001, ns—not significant.

The examination of miRNA hydrolyzing activity involved the utilization of oligonucleotide sequences labeled with fluorescent tags, specifically designed to correspond to the tested miRNAs and distinguished by the prefix “Flu”. Oligonucleotide hydrolysis products were subjected to separation via a 20% polyacrylamide gel electrophoresis. Antibody activity in substrate hydrolysis was calculated by the decrease in the relative amount of the initial substrate in the reaction mixture compared to the control without IgG. The plasma antibodies of the patients were subjected to incubation with Flu-miRNAs. The nonparametric Kruskal–Wallis test (Figure 2) was utilized to evaluate the differentiation among the three patient groups.

The IgG preparations of the Lon group patients demonstrated higher Flu-miR-378a-3p (*p* < 0.05) and lower Flu-miR-146b-5p, Flu-miR-148a-3p (*p* < 0.001), and Flu-miR-766-3p (*p* < 0.0001) hydrolysis activity compared to the control group of patients. Antibody preparations from Cov patients hydrolyzed Flu-miR-4742-3p and Flu-miR-142-3p (*p* < 0.001) with higher activity and hydrolyzed Flu-miR-148a-3p (*p* < 0.0001) with lower activity compared to the control group of patients.

## 3. Discussion

Although miRNAs have attracted attention as potential biomarkers, their practical use in diagnostics has faced obstacles. With miRNAs being short sequences that exhibit a significant degree of similarity [85], a high-specificity method is required for accurately quantifying each miRNA. Currently, the RT-qPCR method is considered the most reliable technique for detecting miRNA. The sensitivity of RT-qPCR is remarkable, allowing for the detection of miRNA molecules at the single nucleotide level, even when present in very low concentrations in the attomolar range [86,87]. Typically, the quantitative analysis of RNA by RT-qPCR involves a two-step procedure: (i) Reverse transcription (RT) employed to create complementary DNA from the initial RNA and (ii) subsequent amplification of the DNA by PCR. The amplification process is monitored in real time using a specific dye (e.g., SYBR Green I) or a specific fluorescent probe. RT-qPCR was initially developed to quantitatively analyze long RNA sequences. This involved using PCR primers that are typically 20 bases long, equivalent to the size of a full-length miRNA. The issue was effectively addressed through the implementation of stem-loop (SL) primers [87]. SL primers enable reliable analysis of miRNAs at low concentrations, enhance result accuracy by ensuring sequence specificity, and mitigate the influence of genomic DNA contaminants. The accuracy of the method is greatly enhanced by its resistance to contaminants [87,88]. In this study, we used this method to determine the concentrations of miRNAs in the plasma of patients, with the focus on convalescent individuals. Throughout the period of recovery, a complex mechanism is implemented to control inflammatory processes, with miRNA serving as a single component of the overall puzzle. We detected an alteration in the expression of three miRNAs out of 10 miRNAs, namely, miR-200c-3p, miR-766-3p, and miR-142-3p. It is worth mentioning that among the patients experiencing rheumatologic complications of long COVID, a notable decrease in the expression of these miRNAs was observed. 

One potential method for reducing miRNA concentrations is antibody-mediated hydrolysis. In addition to their ability to recognize specific substrates, antibodies with catalytic functions can also hydrolyze the substrate [89]. Hence, the isolation of IgG from the blood plasma of patients was followed by an assessment of their catalytic activity in the degradation of fluorescently labeled oligonucleotides corresponding to ten miRNAs. The analysis of antibody catalytic activity revealed that patients in the Lon group exhibited reduced activity in hydrolyzing Flu-miR-146b-5p, Flu-miR-766-3p, Flu-miR-4742-3p, and Flu-miR-142-3p sequences, but increased activity against Flu-miR-378a-3p compared to other patient groups. Lower concentrations of miRNAs correspond to lower activity of antibodies in miRNA degradation. Thus, changes in miRNA concentrations are not associated with their hydrolysis by antibodies, but are regulated by more intricate mechanisms. There is reason to believe that a decrease in the concentration of miRNAs in blood plasma is associated with a decrease in the concentration of corresponding antibodies with miRNA-hydrolyzing activity.

**Figure 3:**
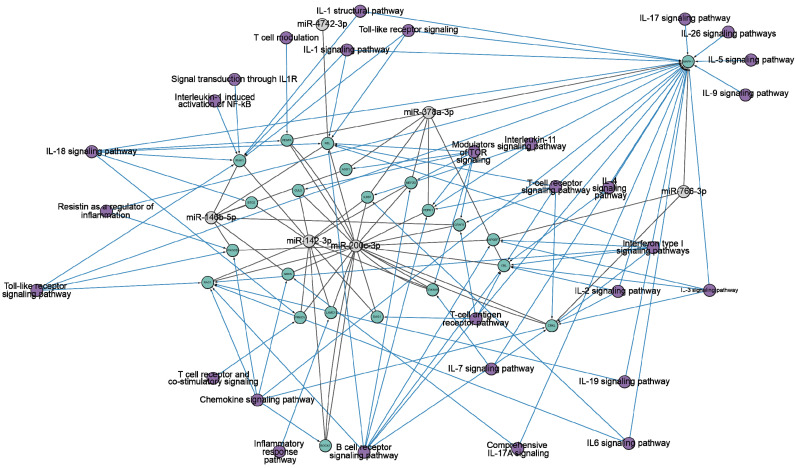
The network diagram of the interactions between target genes (highlighted in green) and six miRNAs (miR-200c-3p, miR-766-3p, miR-142-3p, miR-146b-5p, miR-4742-3p, miR-378a-3p) (highlighted in gray). The information is based on miRTarBase [90] and TargetScan [91] (accessed on 15 June 2024). The representation is limited to genes that regulate the immune response. Purple indicates the ontology of genes. The network is visualized using Cytoscape version 3.10.2.

We hypothesized that changes in miRNA concentrations and antibody-mediated hydrolysis of miRNAs have a complex regulatory mechanism associated with gene pathways concerned with the immune system. Consequently, we conducted a more in-depth analysis of the target genes and pathways related to miR-200c-3p, miR-766-3p, miR-142-3p, miR-146b-5p, miR-4742-3p, and miR-378a-3p. The analysis framework is outlined in Figure S1 (Supplementary Materials). We searched for specific miRNA genes in the miRTarBase [90] and TargetScan [91] databases (accessed on 16 June 2024). Interactions between six specific miRNAs were observed with 1621 genes in the miRTarBase database and 1702 genes in the TargetScan database. Further analysis was conducted on a set of 338 common target genes, which were selected based on their association with six miRNAs. Then, gene-pathway associations were analyzed using the WikiPathways database [92,93] and pathways related to immune response regulation were selected (Figure 3).

Figure 3 clearly demonstrates the impact of all miRNAs on pathways related to the immune response. To illustrate, miR-200c-3p effectively targets 18 genes that are associated with a wide range of signaling pathways, including IL-1, IL-2, IL-3, IL-4, IL-6, IL-7, IL-18, IL-19, interferon type I, chemokine, T-cell, B-cell, and toll-like receptors signaling pathways. Consequently, the scientific literature has noted an increase in the expression of miR-200c-3p in viral infections, including influenza A [94]. miR-200c-3p is involved in the regulation of angiotensin-converting enzyme II (ACE2). ACE2 has many physiological functions in lung tissue, among which it physiologically hydrolyzes angiotensin II [95,96,97]. Overexpression of miR-200c-3p leads to increased angiotensin II concentrations [98]. It has proinflammatory and pro-oxidant effects, which causes vasoconstriction, increased inflammation, thrombosis, and increased collagen synthesis in lung fibroblasts [99,100]. This leads to acute lung injury, alveolar edema, and increased incidence of pulmonary fibrosis [101,102]. Pulmonary fibrosis is a well-known long-term complication of COVID-19, which may be caused by the same mechanism. It was shown that miR-200c-3p expression level increases with increasing disease severity in COVID-19 patients [78,79].

miR-142-3p is associated with 11 genes that influence the immune response through IL-1, IL-7, IL-11, IL-18, chemokine, T- and B-cell, toll-like receptor signaling pathways. miR-142-3p is an evolutionarily conserved vertebrate miRNA that exhibits expression in a variety of hematopoietic cells, including dendritic cells, monocytes, T cells, and B cells [103,104]. miR-142-3p plays an important role in the modulation of immune responses [105,106]. It was shown that miR-142-3p is part of a detrimental regulatory axis with proinflammatory cytokines IL-1β [107,108] and IL-6 [109] in COVID-19 patients, inducing a response associated with respiratory failure and death. miR-142 regulates the expression of occludin, which affects endothelial permeability [110,111,112], contributing to endothelial dysfunction [113,114,115]. This leads to thromboembolic events [116,117] in patients with severe COVID-19 [118,119,120].

miR-146b-5p, miR-378a-3p, and miR-766-3p are found to be associated with 7, 7, and 3 genes, respectively, and are known to be connected to diverse pathways involved in the immune response. Although miR-4742-3p is associated with just one gene, it shares common associations with IL-1, IL-18, IL-19, interferon type I, T- and B-cell, and toll-like receptor signaling pathways, just like the other miRNAs being considered. MiR-146b-5p and miR-378a-3p have been described as a tumor suppressor and anti-inflammatory effector [121,122]. Furthermore, the computational analysis determined the capacity of miR-4742-3p to bind to the RNA of SARS-CoV-2 and hinder the production of viral proteins [123].

In conclusion, our findings have revealed the changes in the expression of three specific miRNAs (miR-200c-3p, miR-766-3p, miR-142-3p) among COVID-19 survivors and individuals with long-COVID-related rheumatologic issues. These patients exhibited changes in the antibody-mediated hydrolysis activity of five specific miRNAs (Flu-miR-146b-5p, Flu-miR-378a-3p, Flu-miR-766-3p, Flu-miR-4742-3p, and Flu-miR-142-3p). Viral infection activates or represses the expression of cellular miRNAs, which in turn modulate the host response to infections [124]. MiRNAs are post-transcriptional regulators of gene expression. They target specific mRNA sequences and control protein production by binding to the untranslated region of the mRNA [125]. Many miRNAs are associated with the regulation of a large number of pathways. The bioinformatics methods allow us to identify the genes that miRNAs influence. In this work, we have shown that all miRNAs participate in a complex network of interactions. Numerous signaling pathways related to the immune system function have been associated with all these miRNAs. The study of the interplay between miRNAs and immune reactions in organisms holds significance in the field of modern biomedicine. It has the potential to greatly enhance our understanding of pathogenesis mechanisms, facilitate the development of diagnostic biomarkers, and inform treatment strategies for the consequences of not only COVID-19 but also other viral infections.

## 4. Materials and Methods

### 4.1. Donors and Patients

This study protocol underwent a thorough review and received approval from the local ethical committee at the ICBFM SB RAS, (the protocol of 15 August 2021). In accordance with the Helsinki Ethics Committee’s recommendations, patients and healthy donors were duly informed and gave consent for their blood donation for scientific purposes.

Venous blood was collected on an empty stomach using vacuum tubes that contained coagulation activators. The blood samples in the tubes underwent centrifugation at 3000× *g* for 10 min using a refrigerated Centrifuge 5810. The resulting plasma, which had been separated from red blood cells, was divided into 1 mL aliquots and stored at a temperature of −70 °C.

The patients involved in this study were categorized into 3 study groups: 

Cov refers to patients who successfully recovered from COVID-19 without any complaints, with a total number of 24 donors. The blood plasma of the Cov group patients was collected in 2020–2021. 

Lon refers to patients who experienced persistent rheumatologic symptoms alongside COVID-19. The Lon group was formed in 2021, with a total number of 16 donors. The patients were examined by a rheumatologist in dynamics, with most patients in this group complaining of joint and muscle pain (n = 16), fever to subfebrile digits (n = 15), fatigue and weakness (n = 13), shooting pains in the body (n = 7), increased anxiety related to their condition (n = 6), and numbness of extremities (n = 5).

Neg refers to conditionally healthy donors who did not have COVID-19, with a total number of 18 donors.

The characteristics of the study groups of patients are presented in Table S1. The composition of the groups was adjusted to ensure gender and age parity. ELISA was used to confirm the presence of persistent SARS-CoV-2 infection and the absence of infection in the control group, targeting the S- and N-proteins of the virus [53,126].

### 4.2. Isolation of miRNAs

MiRNA isolation from blood plasma samples was performed using the reagents from the “Total RNA and miRNA isolation kit” (LRU-100-50, Biolabmix, Russia, Novosibirsk) according to the manufacturer’s instructions. The isolated miRNA pool was stored in low sorption tubes at −20 °C. The RNA concentration was determined spectrophotometrically by measuring the absorbance at 260 nm.

### 4.3. Reverse Transcription Using SL-Primers

The reverse transcription reaction was performed using the OT kit M-MuLV-RH (Biolabmix, Novosibirsk, Russia) in combination with a specific SL-primer (Table 1). The reaction mixture comprised 2 μL of SL-primer (1 μM), 2 μL of the miRNA being tested, and 8 μL of DEPC pretreated water. The mixture was heated at 70 °C for 3 min to disrupt secondary structures and immediately cooled in ice. The subsequent addition involved a reaction mixture composed of 4 μL of 5 × OT buffer, 1 μL of M-MuLV-RH revertase (concentration: 10 units/μL), and 3 μL of DEPK-pretreated water. 

Reverse transcription was performed in 45 cycles according to the following protocol: 

1. 30 min at 16 °C;

2. (30 s at 30 °C, 30 s at 42 °C, 1 s at 50 °C) × 45;

3. 5 min at 85 °C.

### 4.4. Real-Time PCR

The cDNA generated by reverse transcription was analyzed using the BioMaster HS-qPCR PCR kit (Biolabmix, Novosibirsk, Russia). The reaction mixture (20 µL) contained 10 µL of BioMaster HS-qPCR (2×), 0.4 µL of forward primer (10 µM), 0.4 µL of universal reverse primer SL_rev (Table 2), 0.2 µL of universal probe with FAM dye (10 µM), 2 µL of cDNA (SL-OT product), and 7 µL of sterile water.

The amplification procedure included the following steps: 5 min at 95 °C, 45 cycles (20 s at 95 °C and 1 min at 55 °C). The fluorescent signal detection was performed through the FAM at the end of each cycle. 

MiRNAs were quantified in the blood plasma using a synthetic miR-148a-3p with known concentration as normalization. A calibration curve was generated using synthetic miRNA at concentrations of 10^–1^ ng/µL, 10^–3^ ng/µL, and 10^–5^ ng/µL. Subsequently, OT-PCR and real-time PCR reactions were conducted. The construction of calibration curves involved the utilization of Microsoft Excel 2016 software and the analysis of four repeated measurements. The equation for the calibration curve was derived using CFX Maestro software version 2.3 (BioRad, Singapore, Tower): y = −2.384 ln(x) − 1.983, with y representing the Cq value obtained post-amplification, and x denoting the concentration.

### 4.5. Identification of IgG Activity in the miRNA Hydrolysis Reaction

IgGs were isolated from the blood plasma of patients using affinity chromatography on Protein-G-Sepharose similar to [127]. The efficiency of miRNA cleavage in 20% PAAG was used to determine the relative catalytic activity of IgG, as described in [58,59]. The reaction mixture (10 µL) contained 20 mM tris–HCl, pH 7.5; IgG preparation (0.1 mg/mL), and fluorescently labeled synthetic miRNA (Table 3), with a concentration from 0.09 to 0.39 f.u./mL (depending on the miRNA). The reaction mixture was incubated for 1 h at 37 °C. A reaction mixture without IgG was used as a control. Following the incubation, the reaction mixture was supplemented with 10 μL of denaturing buffer containing 8 M urea and 0.025% xylenecyanol. The marker (Leader) was procured via a process of limited alkaline hydrolysis, employing a bicarbonate buffer with a concentration of 0.05 M and miRNA concentrations ranging from 0.08 to 0.35 f.u./mL. The mixture was incubated for 15 min at 90 °C. After that, 10 µL of denaturing buffer was added. The hydrolysis products were analyzed using electrophoresis under denaturing conditions (20% acrylamide, 8 M urea, 1× TBE pH 8.3) at 800 V, 40 mA, for 2 h. The hydrolysis products were visualized using the iBright™ CL1500 Imaging System (Invitrogen™, Waltham, MA, USA), and the analysis was performed using the GelAnalyzer 23.1 software.

### 4.6. Statistical Analysis

The calculation of the significance of differences between patient groups was performed utilizing the Mann–Whitney U-test from the Python library SciPy v 1.11.4 [128] and the Kruskal–Wallis criterion from the standard R package v 4.3.2. The correlation coefficients between groups were computed using the nonparametric Spearman rank correlation method available in the Python library SciPy. The *p*-value was used to determine statistical significance, with a minimum threshold set at 0.05.

### 4.7. Target Predictions of the miRNAs Analyzed

The miRTarBase [90] and TargetScan [91] databases were utilized to search for target miRNA genes. This was performed through the CyTargetLinker 4.0.0+ application [129] (accessed on 15 June 2024). Subsequently, we utilized the WikiPathways database [92,93] to perform pathway analysis on the identified genes. Genes associated with the control of the immune response were specifically selected. The interaction network was visualized using Cytoscape 3.10.2.

## Figures and Tables

**Table 1:** SL-primers used in the study.

Name	Nucleotide Sequence
let–7f–5p SL	5′–GTTGGCTCTGGTGCAGGGTCCGAGGTATTCGCACCAGAGCCAACAACTAT–3′
146b–5p SL	5′–GTTGGCTCTGGTGCGGGTCCGAGGTATTGCACCAAGAGCCAACCAGCCT–3′
148a–3p SL	5′–GTTGGCTCTGGTGCGGGTCCGAGGTATTGCACCAAGAGCCAACACAAAG–3′
200c–3p SL	5′–GTTGGCTCTGGTGCGGGTCCGAGGTATTGCACCAAGAGCCAACCTCCATC–3′
378a–3p SL	5′–GTTGGCTCTGGTGCGGGTCCGAGGTATTGCACCAAGAGCCAACGCCTTC–3′
9–5p SL	5′–GTTGGCTCTGGTGCAGGGTCCGAGGTATTCGCACCAGAGCCAACTCATAC–3′
766–3p SL	5′–GTTGGCTCTGGTGCAGGGTCCGAGGTATTCGCACCAGAGCCAACGCTGAG–3′
3125 SL	5′–GTTGGCTCTGGTGCAGGGTCCGAGGTATTCGCACCAGAGCCAACTCTCTC–3′
4742–3p SL	5′–GTTGGCTCTGGTGCAGGGTCCGAGGTATTCGCACCAGAGCCAACCTGCAG–3′
142–3p SL	5′–GTTGGCTCTGGTGCAGGGTCCGAGGTATTCGCACCAGAGCCAACTCCATA–3′

**Table 2:** Primers used in the study.

Name	Nucleotide Sequence	Length in Nucleotides	Melting Point (°C)
let–7f–5p for	5′–GTTTGGTGAGGTAGTAGATTGT–3′	22	51
146b–5p for	5′–GTTTTTCGTGAGAACTGAATTCCAT–3′	25	52.8
148a–3p for	5′–GTTTTGGTCAGTGCACTACAGAA–3′	23	53.5
200c–3p for	5′–GTTTGGTAATACTGCCGGGTAAT–3′	23	53.5
378a–3p for	5′–GTTTTTGACTGGACTTGGAGTCA–3′	23	53.5
9–5p for	5′–GUGGAAGACUUCGAGGCCUUG–3′	22	56.3
766–3p for	5′–GAGCUUGGGAUAGAGGGCUUA–3′	22	54.4
3125 for	5′–GCCAGCUGGAAGUUGAGGAAG–3′	22	56.3
4742–3p for	5′–GCUUAGCUCGUGGUCCCGGAC–3′	21	60.2
142–3p for	5′–UGGAGGAAGAGGUGGAGGAAG–3′	21	56.3
Un rev primer	5′–GTGCAGGGTCCGAGGT–3′	16	51.1

**Table 3:** 5′-Flu-labeled oligoribonucleotides used as substrates in this study.

Flu-miR	Nucleotide Sequence	Length in Nucleotides
Flu-let–7f–5p	5′–Flu–UGAGGUAGUAGAUUGUAUAGUU	22
Flu-146b–5p	5′–Flu–UGAGAACUGAAUUCCAUAGCCUG	23
Flu-148a–3p	5′–Flu–UCAGUGCACUACAGAACUUUGU	22
Flu-200c–3p	5′–Flu–UAAUACUGCCGGGUAAUGAUGGA	23
Flu-378a–3p	5′–Flu–ACUGGACUUGGAGUCAGAAGGC	22
Flu-9–5p	5′–Flu–UCUUUGGUUAUCUAGCUGUAUGA	23
Flu-766–3p	5′–Flu–ACUCCAGCCCCACAGCCUCAGC	22
Flu-3125	5′–Flu–UAGAGGAAGCUGUGGAGAGA	20
Flu-4742–3p	5′–Flu–UCUGUAUUCUCCUUUGCCUGCAG	23
Flu-142–3p	5′–Flu–UGUAGUGUUUCCUACUUUAUGGA	23

## Data Availability

Empirical data that do not relate to the personal data of donors can be provided by Anna Timofeeva upon request.

## Supplementary Materials

The following supporting information can be downloaded at: https://www.mdpi.com/article/10.3390/ncrna10050048/s1, Table S1. Characteristics of the patients. Figure S1: Interaction networks between target genes and six miRNAs.
