# Supplementary material for: Circulating miRNAs in the Plasma of Post-COVID-19 Patients with Typical Recovery and Those with Long-COVID Symptoms: Regulation of Immune Response-Associated Pathways
Source: Noncoding RNA. 2024 Sep 2;10(5):48. doi: 10.3390/ncrna10050048 (PMC11417918; doi:10.3390/ncrna10050048)
Supplement: Supplementary file 1 [file ncrna-10-00048-s001.zip › ncrna-3127604-supplementary.pdf]

non-coding RNA

MDPI

Table S1. Characteristics of the patients. 1

ID	Sex	Date of first symptoms of COVID-19	Date of blood collection	Age	Titer S-IgG*	Rheumatological symptoms
**Lon**						
ZNV	F	11.06.2020	28.02.2021	30	12.4	joint and muscle pain, fever to subfebrile digits, fatigue and weakness, shooting pains in the body, numbness of extremities
AAV	F	26.06.2020	21.03.2021	28	5.3	joint and muscle pain, fever to subfebrile digits
DEM	M	15.07.2020	19.01.2021	23	7.3	joint and muscle pain, fever to subfebrile digits, fatigue and weakness, increased anxiety related to their condition
TOV	F	09.08.2020	22.04.2021	37	8.4	joint and muscle pain, fever to subfebrile digits, fatigue and weakness, shooting pains in the body, increased anxiety related to their condition
KAV	F	01.09.2020	28.04.2021	33	5.5	joint and muscle pain, fever to subfebrile digits, fatigue and weakness
KhGA	M	20.09.2020	09.06.2021	57	13.5	joint and muscle pain, increased anxiety related to their condition
DYuH	F	10.10.2020	16.06.2021	39	10.5	joint and muscle pain, fever to subfebrile digits, fatigue and weakness, shooting pains in the body, increased anxiety related to their condition
KKS	F	01.11.2020	3.06.2021	37	8.5	joint and muscle pain, fever to subfebrile digits
IVG	F	06.12.2020	22.07.2021	31	10.7	joint and muscle pain, fever to subfebrile digits, fatigue and weakness, shooting pains in the body, numbness of extremities
SOA	F	18.12.2020	13.09.2021	36	12.5	joint and muscle pain, fever to subfebrile digits, fatigue and weakness, increased anxiety related to their condition
LNV	F	13.02.2021	12.10.2021	46	5.4	joint and muscle pain, fever to subfebrile digits, fatigue and weakness, shooting pains in the body, numbness of extremities
GDA	M	04.03.2021	07.10.2021	41	11.7	joint and muscle pain, fever to subfebrile digits, fatigue and weakness, shooting pains in the body, numbness of extremities
GEB	F	15.03.2021	14.10.2021	33	4.9	joint and muscle pain, fever to subfebrile digits, fatigue and weakness, increased anxiety related to their condition
REI	F	06.04.2021	14.09.2021	31	6.3	joint and muscle pain, fever to subfebrile digits, fatigue and weakness
KEB	F	17.06.2021	19.10.2021	50	8.7	joint and muscle pain, fever to subfebrile digits, fatigue and weakness, shooting pains in the body, numbness of extremities
VAV	M	24.06.2020	05.01.2021	42	10.5	joint and muscle pain, fever to subfebrile digits, fatigue and weakness, increased anxiety related to their condition
**Cov**						
1002	F	27.06.2020	07.10.2020	36	7.1	no
1003	F	28.06.2020	05.10.2020	42	7.9	no
1004	F	04.07.2020	05.10.2020	43	8.5	no
1006	F	19.07.2020	05.12.2020	41	5.1	no
1106	M	23.09.2020	25.12.2020	36	12	no
1117	F	05.10.2020	02.03.2021	51	8.7	no
1120	F	06.10.2020	02.03.2021	35	7.4	no
1121	F	08.10.2020	02.03.2021	30	8.4	no
1123	F	13.10.2020	02.03.2021	49	7.3	no
1126	F	15.09.2020	02.03.2021	50	8.1	no
1151	M	13.10.2020	23.01.2021	36	13.4	no

1166	M	14.10.2020	09.02.2021	21	7.8	no
1171	M	19.10.2020	19.02.2021	42	10	no
1215	F	20.10.2020	02.03.2021	57	8.1	no
1225	F	01.09.2020	04.02.2021	52	12.4	no
1234	F	08.11.2020	04.03.2021	25	11.6	no
1239	M	25.11.2020	14.03.2021	47	13.2	no
1248	F	05.12.2020	16.04.2021	32	8.3	no
1293	M	08.11.2020	19.04.2021	37	7.5	no
1296	F	16.12.2020	19.04.2021	28	10.6	no
1302	F	15.01.2021	26.04.2021	45	7.3	no
1325	F	15.02.2021	25.05.2021	54	8.4	no
1354	F	17.02.2021	24.06.2021	24	7.7	no
1375	F	26.02.2021	02.07.2021	22	8.8	no
**Neg**						
0104	F	no	15.02.2020	25	0.3	no
0106	F	no	15.02.2020	36	0.4	no
0107	F	no	15.02.2020	24	0.3	no
0111	F	no	15.02.2020	25	0.2	no
0112	F	no	15.02.2020	39	0.2	no
0126	F	no	06.03.2020	54	0.2	no
0128	M	no	06.03.2020	30	0.5	no
0131	F	no	06.03.2020	36	0.3	no
0136	F	no	10.03.2020	50	0.5	no
0137	M	no	10.03.2020	42	0.4	no
0145	M	no	10.03.2020	55	0.3	no
0148	M	no	10.03.2020	45	0.2	no
0149	F	no	26.03.2020	41	0.2	no
0156	F	no	26.03.2020	40	0.2	no
0158	M	no	26.03.2020	35	0.3	no
0163	M	no	26.03.2020	45	0.4	no
0175	F	no	16.04.2020	26	0.2	no
0176	F	no	16.04.2020	38	0.3	no

* The serology of SARS-CoV-2 IgG against the S-protein and N-protein of SARS-CoV-2 was evaluated using ELISA Antigma G (Generium, Vladimir, Russia) according to the manufacturer's instructions. A value less than 1.0 was considered negative.

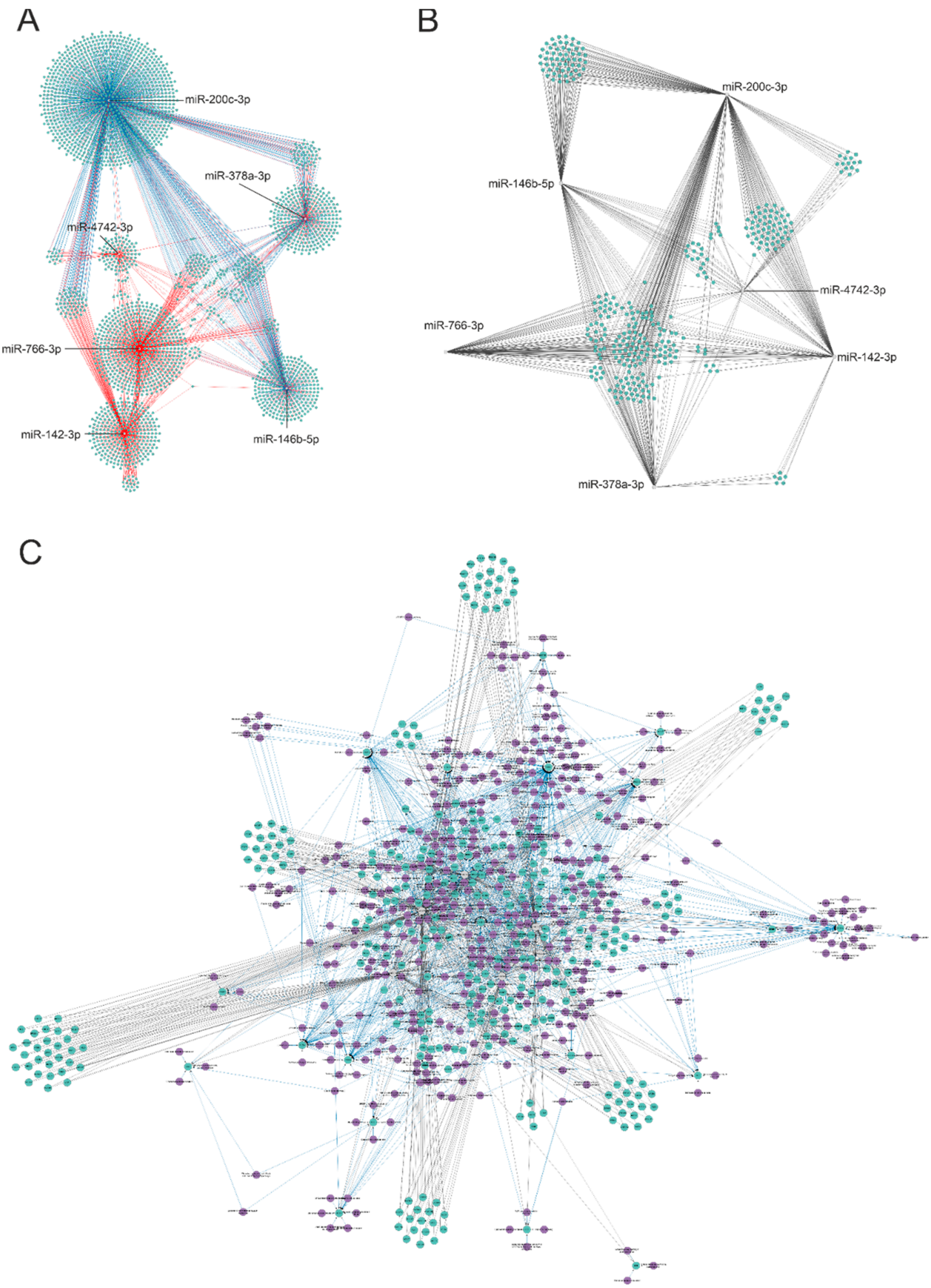

Figure S1. A. Interaction networks between target genes and six miRNAs (miR-200c-3p, miR-766-3p, miR-142-3p, miR-146b-5p, miR-4742-3p, and miR-378a-3p) based on miRTarBase (red edges) and TargetScan (blue edges) (accessed on 15.06.2024). B. Only common interactions between the investigated miRNAs were selected from the total interaction network. C. The gene-pathway association network was then compiled using the WikiPathways database (accessed on 18.06.2024). Gene-targets were visualized using Cytoscape 3.10.2 and CyTargetLinker 4.0.0+. MiRNAs are marked in gray, target genes in green, and pathways in purple.
